# Supplementary figures and images for: On the influence of prior information evaluated by fully Bayesian criteria in a personalized whole-brain model of epilepsy spread
Source: PLoS Comput Biol. 2021 Jul 14;17(7):e1009129. doi: 10.1371/journal.pcbi.1009129 (PMC8312957; doi:10.1371/journal.pcbi.1009129)

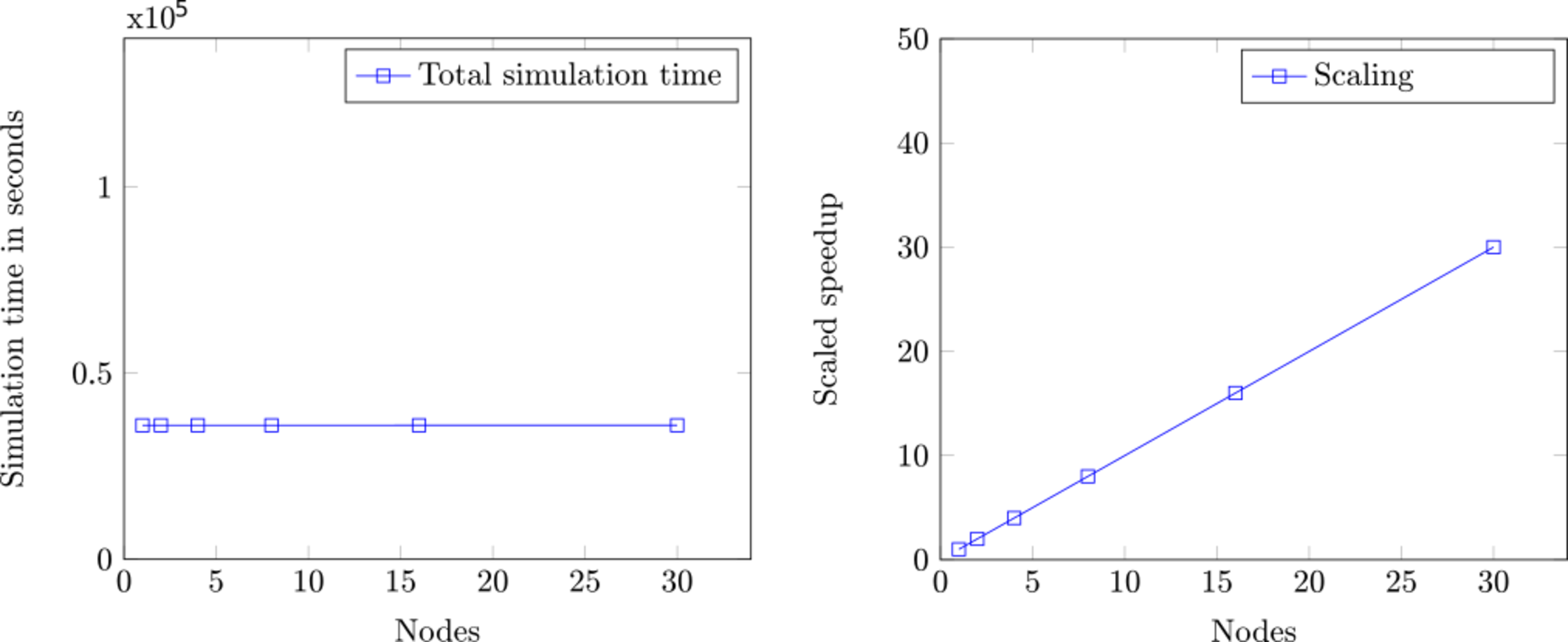

Supplement: S1 Fig — (A) The total simulation time remains constant even when the number of Markov chains increases. (B) The speedup achieved increases linearly with the number of computing units. (TIF) [file pcbi.1009129.s001.tif]

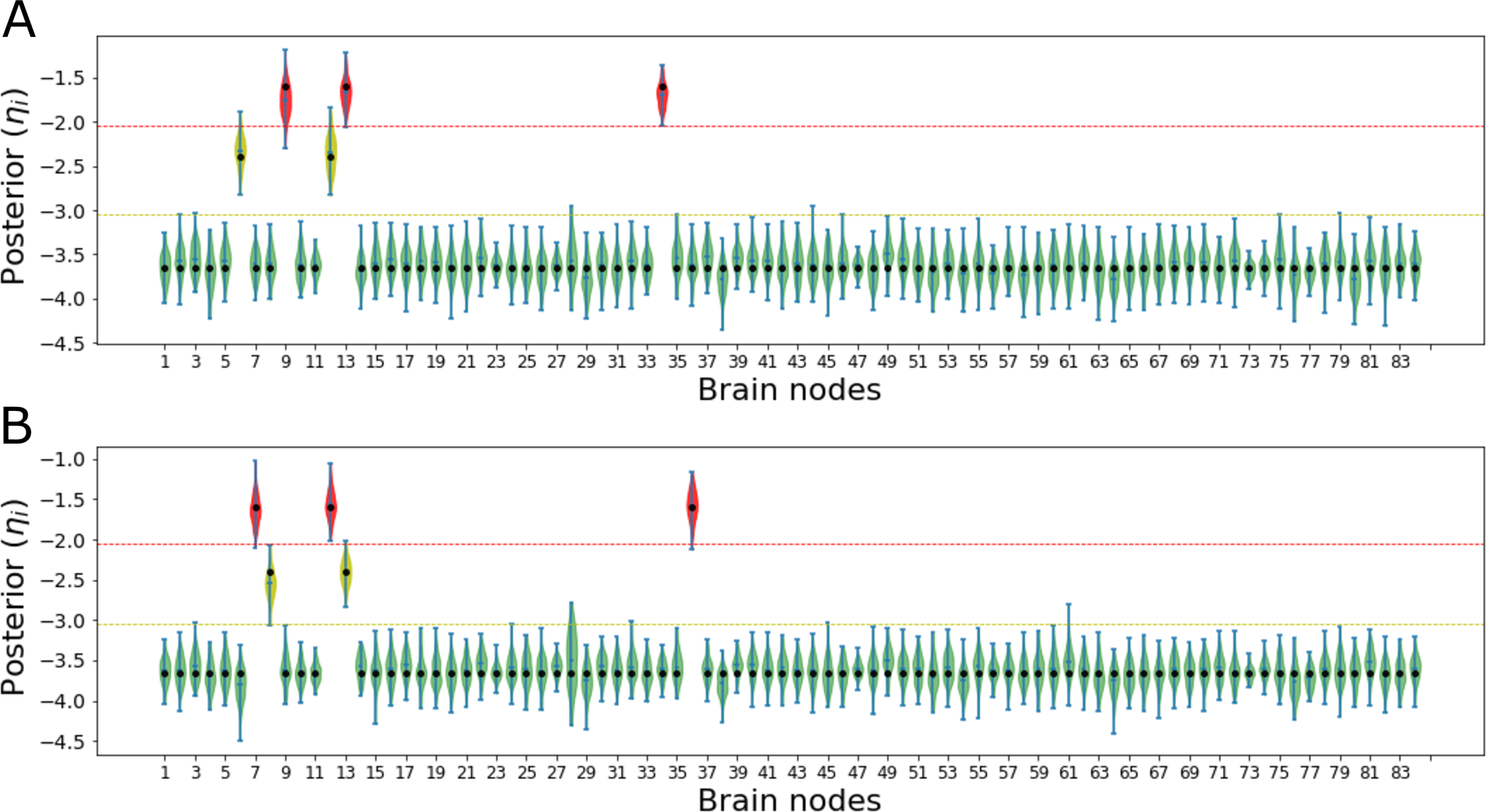

Supplement: S2 Fig — Violin plots of the estimated densities of excitability parameters for 84 brain regions in two different analyses. To simulate the seizure activity for a virtual patient, the excitability value was chosen as ηez = −1.6 corresponding to regions in EZ, and ηpz = −2.4 for the regions in PZ, whereas all the other regions were defined as HZ with ηhz = −3.6. (A) Three brain regions are selected as part of EZ at the nodes EZidx ∈ {9, 13, 34}, and two regions as PZ at the nodes PZidx ∈ {6, 12}. (B) Two brain regions are selected as part of EZ at the nodes EZidx ∈ {7, 12, 36}, and two regions as PZ at the nodes PZidx ∈ {8, 13}. The ground truth values are displayed by the filled black circles. (TIF) [file pcbi.1009129.s002.tif]

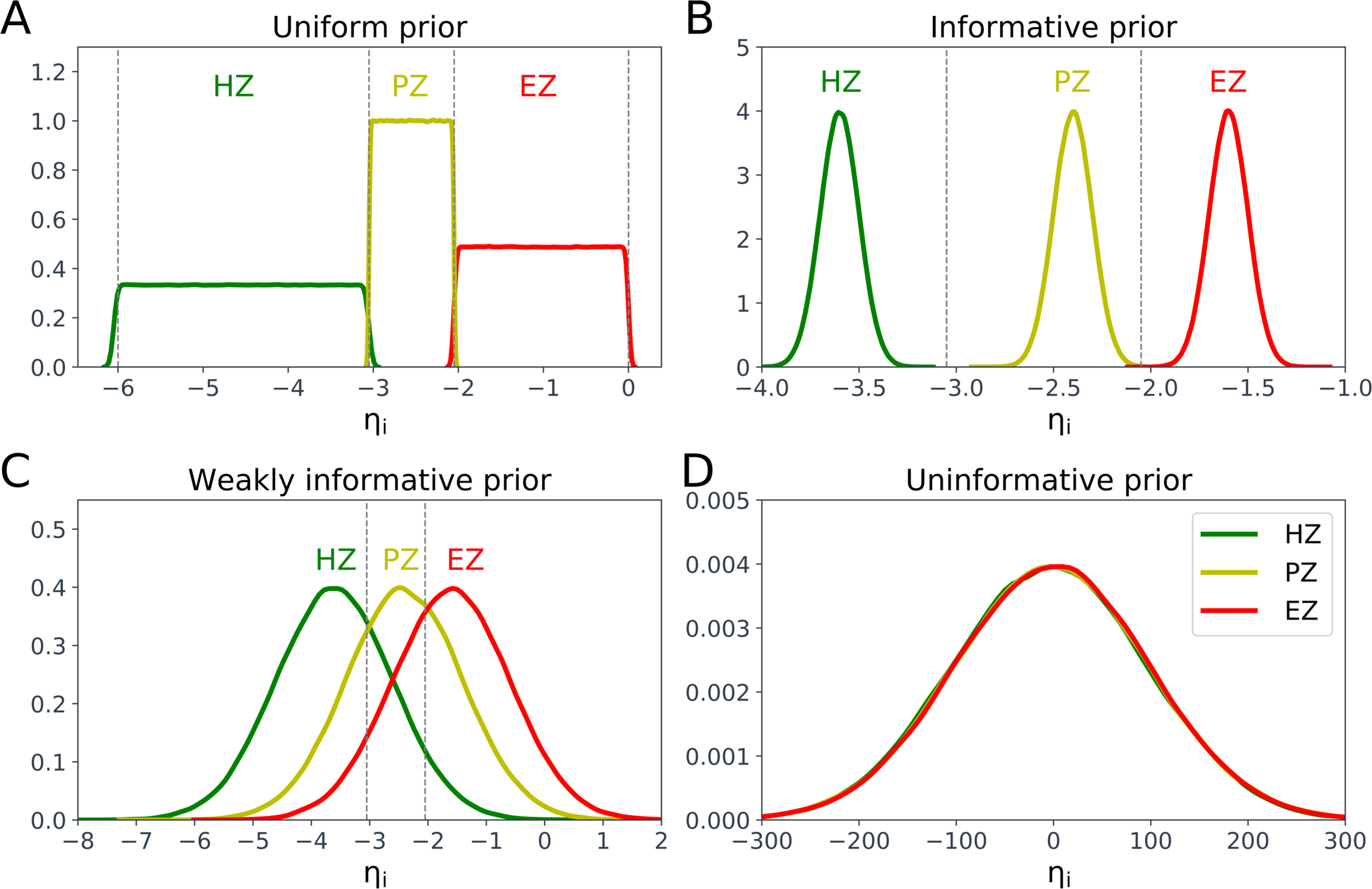

Supplement: S3 Fig — (A) The uniform prior truncated in the ranges [a, ηc − Δη], [ηc − Δη, ηc], and [ηc, b] corresponding to HZ, PZ, and EZ, respectively. Here, ηc = −2.05, Δη = 1.0, a = −6.0, b = 0.0. (B) Informative prior defined by N(μhypo,σ2), where μez = −1.6, μpz = −2.4, and μhz = −3.6 correspond to EZ, PZ, and HZ hypotheses, respectively, whereas σ implies our information about the epileptogenicity hypothesis. Here, informative prior with σ = 0.01. (C) Weakly informative prior with σ = 1.0 (D) Uninformative prior with σ = 1000. (TIF) [file pcbi.1009129.s003.tif]

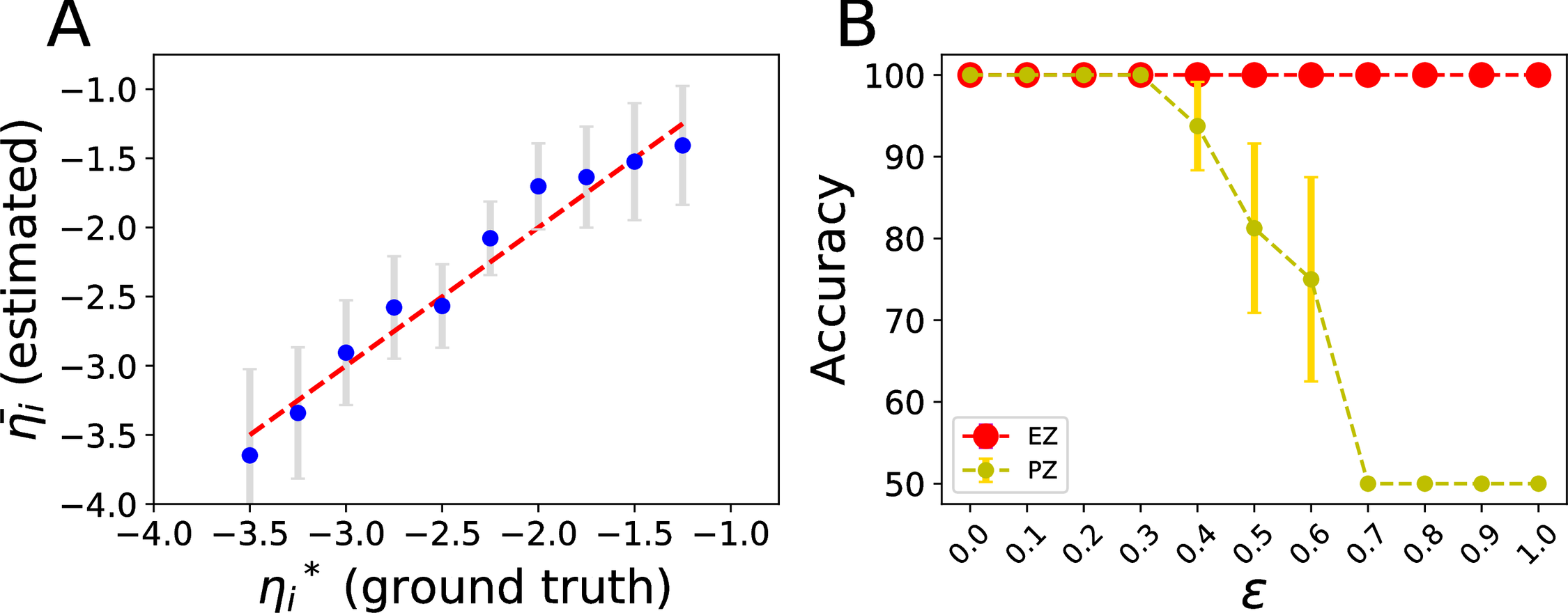

Supplement: S4 Fig — (A) Excitability values used for simulation (ηi*) versus the estimated values (ηi¯) for a selected brain region (node number 6). As the node dynamics are varied by changing the excitability parameter (functional component), the model inversion by NUTS algorithm demonstrates an accurate and robust estimation by recovering the ground truth. Dashed red line represents a perfect fit. (B) The classification accuracy in EZ and PZ prediction as the value of structural connections (SC) to the selected brain region is decreased from 100% to 0% of value used in simulation (SC*), i.e. SC = (1 − ∊)SC*. As the dynamics in EZ depend only on the excitability parameter, the EZ prediction remains accurate irrespective of changes in SC (shown in red). However, the seizure propagation depends also on the network properties, thus the model performance in PZ prediction depends critically on the strength of connections to these nodes (shown in yellow). (TIF) [file pcbi.1009129.s004.tif]

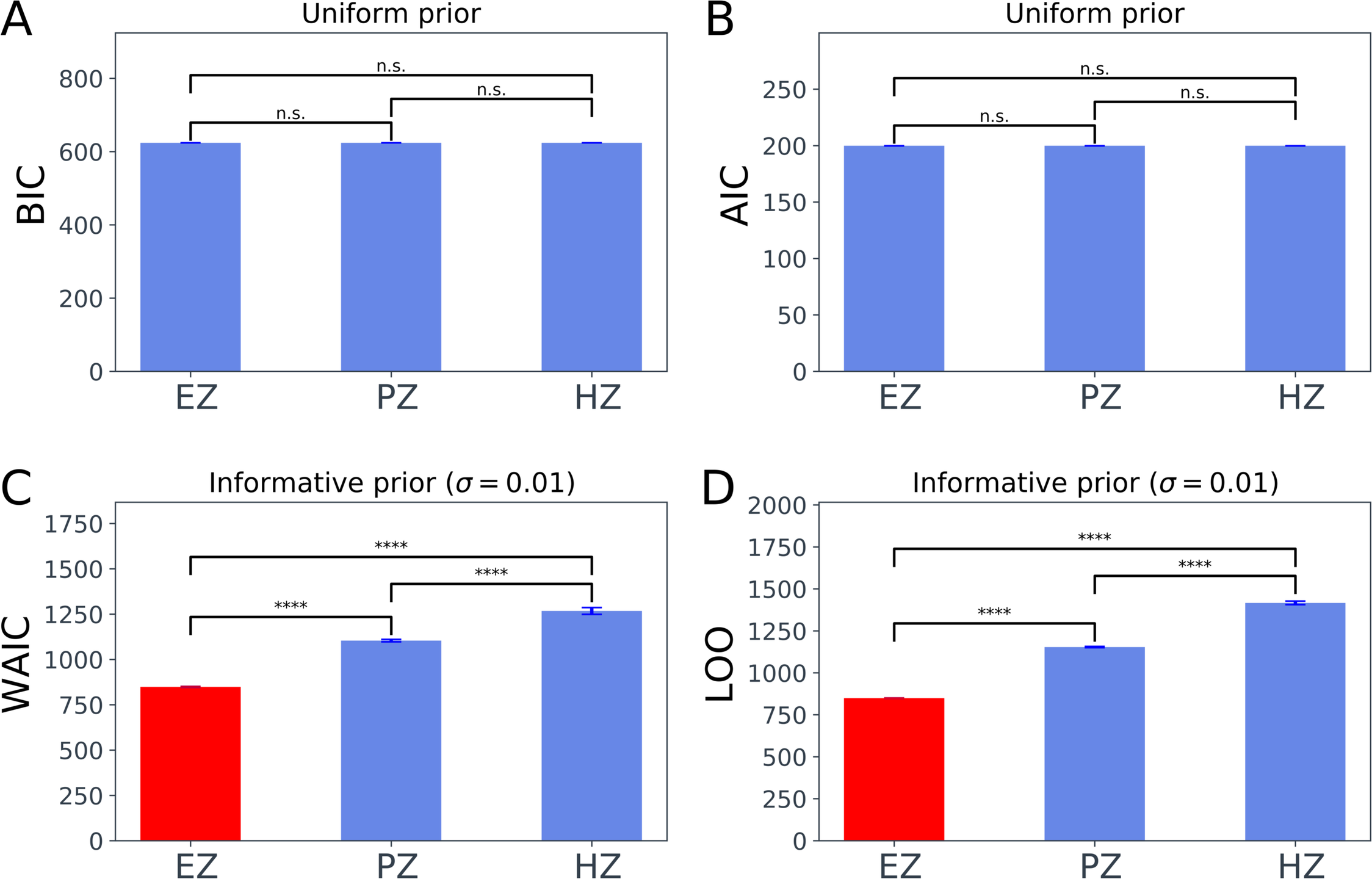

Supplement: S5 Fig — Here, the brain node with the highest excitability value corresponding to a region in EZ was analyzed, and each of the EZ, PZ, and HZ hypotheses was averaged over 100 estimations to determine whether the computed information criterion is statistically significant for the true hypothesis. Panels (A) and (B) show BIC and AIC, respectively, computed for three EZ, PZ, and HZ hypotheses, while the ground truth of excitability parameter η in the simulation was -1.5 (i.e., a brain region corresponding to EZ). Placing a uniform prior on each hypothesis, both BIC and AIC are averaged over 100 MLL estimations. In both BIC and AIC, there is no significant difference (n.s.) between different hypotheses. (C) and (D) show the computed WAIC and LOO, respectively, for three EZ, PZ, and HZ hypotheses. Here, we have placed an informative prior on the excitability parameter η by using Gaussian distribution N(μhypo,σ2), where σ = 0.01, and μez = −1.5, μpz = −2.5, μhz = −3.5, corresponding to EZ, PZ, and HZ hypotheses, respectively. Each bar plot shows the average over 100 HMC chains randomly initialized in search space. In contrary to classical information criteria, both WAIC and LOO correctly favor the true hypothesis with a high level of statistical significance (EZ hypothesis as shown in red, ****p ≤ 0.0001). Moreover, it can be seen that WAIC closely approximates LOO cross-validation. (TIF) [file pcbi.1009129.s005.tif]
