# Supplementary material for: On the influence of prior information evaluated by fully Bayesian criteria in a personalized whole-brain model of epilepsy spread
Source: PLoS Comput Biol. 2021 Jul 14;17(7):e1009129. doi: 10.1371/journal.pcbi.1009129 (PMC8312957; doi:10.1371/journal.pcbi.1009129)
Supplement: Attachment — Submitted filename: BVEP_prior_PLOSCB_ReviewereRsponses_R2.pdf [file pcbi.1009129.s007.pdf]

Manuscript: COMPBIOL-D-20-01623

Title: On the influence of prior information evaluated by fully Bayesian criteria in a personalized whole-brain model of epilepsy spread

for consideration at PLOS Computational Biology.

Corresponding Author: Meysam Hashemi, Viktor K. Jirsa

Co-Authors: Anirudh N. Vattikonda, Viktor Sip, Sandra Diaz-Pier, Alexander Peyser, HuifangWang, Maxime Guye, Fabrice Bartolomei, Marmaduke M. Woodman

The authors thank the reviewers for their insightful and constructive comments to improve the paper. We have now addressed the comments of Reviewer #2 in our revised manuscript. The changes in the manuscript have been marked in red color.

Response to Reviewer #2:

We thank the reviewer for your review and the very constructive comments.

Reviewer #2: I appreciate the authors genuine efforts to engage with my earlier comments and the revised manuscript is certainly improved in my view. My remaining comments are:

"We thank the reviewer for this comment, as the deployment on actual human data is indeed our motivation. We do not agree though that the matched ground truth model is straight forward to work. The Bayesian inversion of the reduced model is a very challenging task and to the best of our knowledge, there is no similar high-dimensional problem with nonlinear generative model in the literature of brain network modeling."

I was not suggesting that the Bayesian inversion is an easy task. I recognize that even the 2D model involves many parameters and so the inference problem is not trivial at all. My only point was that the final outcome -- that the inference led to `correct' inference of excitability -- was expected given that the 2D and 6D models are essentially matched in their underlying dynamics. I generally feel that the authors revisions help make this point more transparent.

We agree with reviewer that the seizure initiation and propagation are invariant under model reduction as the main motivation here was to reduce the computation time regarding model inversion while the spatial map of excitability is preserved. This point was already highlighted in the revision.

“Thank you for mentioning this point. In order to avoid the non-identifiability for fitting the SEEG data, the contacts are selected according to the mean energy of bipolars to provide a bijection map between source activity (generated by Epileptor) at brain regions and measurement at electrodes. This point is now added in a subsection Stereotactic-EEG (SEEG) data preprocessing in Material/Methods. In addition, the codes and documentation for SEEG preprocessing is added in Github repository.”

I am still not convinced that this part of the paper has a point. The veracity of the Bayesian framework has already been established by this point in the paper, so the only reason to go through this would be to make a statement about the ability of the method to localize seizure foci in actual brains (there is no additional technical validation offered by this example). However, as the authors point out, this validation is beyond the scope of the paper. I think the authors need to be clear about what this example provides if it is not a validity study regarding the ability of the method to provide inferences that agree with clinical assessments.

As said by both, the reviewer and us, the veracity of the technical framework has been established and the empirical example is not aimed at the validation of actual patient data. Nevertheless, empirical biological data have a large range of spatial and temporal variability of many heterogeneous multiscale sources, which cannot be straightforwardly mimicked in simulated data. The use of The Virtual Brain platform is one community attempt of capturing some of this variability but does not replace the full-fledged biological complexity. The empirical use case in our paper is aimed at providing an illustrative example, demonstrating the principle of feasibility, and providing concreteness. Both tends to aid better communication of methodological results, which is typically appreciated by the less technical reader. This is now clarified in the Discussion section:

“In this study, we focused on synthetic data to validate the technical reliability of our approach, the self-tuning sampling algorithms (NUTS), and more importantly, the fully Bayesian information criteria and systematic cross-validation for measuring the out-of-sample prediction accuracy. Showing the estimations for a patient cohort requires a detailed non-trivial comparison with the clinical evaluations and outcome after surgery, involving significant other work such as group analysis (in particular homogenization of cohort), which was not aimed in this study. This remains to be critically investigated in future work. However, in order to demonstrate how the proposed approach can be applied to empirical data we have shown an illustrative example in Fig. 7.”

“The expression “virtual brain” has become a commonly, albeit loosely, used terminology for full brain modeling using connectomes and neural mass models at network nodes. Historically, this derives from the use of the neuroinformatics platform The Virtual Brain (https://www.thevirtualbrain.org)”

I must disagree that this is common terminology. I have no issue with the nickname “the virtual brain”. However, this is nonetheless a nickname and it is inappropriate for the authors to attempt to project this as a standard term for all dynamical models of brain networks. “virtual brain” should be used in quotes with citation and qualification as to what this term actually means.

By no means we wanted to imply that “virtual brains” is a standard term for all dynamical models of brain networks. It is not. The expression is however regularly used when making

reference to full brain network models that are informed by DTI-derived structural connectivity data. To avoid any further misunderstanding, we have now changed the terminology from “virtual brain modeling” to “whole-brain modeling”, which is also commonly used.

In Introduction section, we have also added:

The Virtual Brain (TVB) is a computational framework to simulate large-scale brain network models based on individual subject data (Sanz-Leon et al. 2013, Sanz-Leon et al. 2015). TVB is designed to simulate collective whole brain dynamics by virtualizing brain structure and function, allowing simultaneous outputs of a number of experimental modalities. This open-source neuroinformatics platform has been extensively used to simulate common neuroimaging signals including functional MRI (fMRI), EEG, SEEG and MEG with a wide range of clinical applications from Alzheimer disease (Zimmermann et al 2018), chronic stroke (Falcon et al. 2016) to human focal epilepsy (Jirsa et al. 2016).
