# Supplementary material for: On the influence of prior information evaluated by fully Bayesian criteria in a personalized whole-brain model of epilepsy spread
Source: PLoS Comput Biol. 2021 Jul 14;17(7):e1009129. doi: 10.1371/journal.pcbi.1009129 (PMC8312957; doi:10.1371/journal.pcbi.1009129)
Supplement: Attachment — Submitted filename: BVEP_prior_PLOSCB_ReviewereRsponses.docx [file pcbi.1009129.s006.docx]

Manuscript: COMPBIOL-D-20-01623

**Title**: On the influence of prior information evaluated by fully Bayesian criteria in a personalized virtual brain model of epilepsy spread

for consideration at PLOS Computational Biology.

Corresponding Author: Meysam Hashemi, Viktor K. Jirsa

Co-Authors: Anirudh N. Vattikonda, Viktor Sip, Sandra Diaz-Pier, Alexander Peyser, HuifangWang, Maxime Guye, Fabrice Bartolomei, Marmaduke M. Woodman

**Response to Reviewers**

The authors thank the reviewers for their insightful and constructive comments to improve the paper. We have now addressed the reviewers’ comments in our revised manuscript and provide here below detailed explanations of the changes. The changes in the manuscript have been marked in red color.

Following reviewers’ comments, Introduction has been reorganized. Moreover, three figures have been added: Fig 5 (in current revision) illustrating the performance of the model and limitations in the inference for different level of prior information about the global coupling parameter, and Figures S2, S4 showing the robustness of inference.

Detailed Responses:

**Reviewer #1:** The paper discusses the important aspect of using prior knowledge and data in a bayesian setting for quantitative analysis of presurgical investigations within epilepsy surgery. The main ideas of the paper are presented well and I find no concerns with the data analysis.

As minor issues,

The VEP includes the SC map and a 6D epileptor model at each node. The inversion is done as I understand it using a 2D epilpetor model. What is the reason to include the 6D model instead of the 2D model at each node as both models managed to create the envolope that is used for data inversion.
The inversion is done using a highly non-linear generative model. How brittle is this inversion scheme. It worked fine in the study but the inversion was done on partly tailored data as I understand it.

We are grateful to the reviewer for the interest in our work and for the positive comments.

In this study, we used the 6D Epileptor model, capable to generate the full range of epileptic seizures types (Saggio et al eLife 2020), thus providing realistic seizure data in the forward simulation. This approach has the advantage that we can evaluate the full workflow, and not just the inversion part; furthermore, the presentation will benefit from more proximity to real-life data and thus eliminate an additional abstraction step (the computation of the envelope function) for less technically, but more clinically interested readers. The 2D reduction is used to reduce the computational time in Bayesian inversion since the gradient-based sampling by HMC is computationally expensive. Mathematically speaking, the reduction is able to demonstrate only saddle-node bifurcation out of six types of bifurcations involved in seizure initiation and propagation, but the technique, when applied to data here, applies to all seizure types, but with less efficiency for those other than the saddle-node type. This is acceptable as the former is the most dominant seizure type (about 80%, Saggio et al eLife 2020). To address this question, in revision Material/Methods we have added:

“Note that, the full VEP model comprises five state variables governed by three different timescales (Eq. 1) and is a complete taxonomy of epileptic seizures, which demonstrate a thorough description of system bifurcations that give rise to onset, offset and seizure evolution (Jirsa et al. 2014, Saggio et al. 2020). Whereas, the 2D reduced variant of the model (Eq. 2) is limited to modeling the average of fast discharges during the ictal seizure states and the seizure propagation. This reduction is limited to demonstrate only saddle-node bifurcation out of six types of bifurcation involved in the spatio-temporal seizure dynamics. However, the seizure initiation from EZ and propagation to PZ are invariant under this model reduction (Proix et al. 2014, Proix et al. 2017). The 2D VEP model can successfully predict the key data features such as onset, propagation envelope and offset, while considerably alleviating the computational time of the Bayesian inversion (Jirsa et al. 2017, Hashemi et al. 2020).”

In addition, we have added one figure in the revision (now Fig 5) to demonstrate the performance of model and the limitation in Bayesian inversion. In this figure, we have shown how the accuracy, z_scores, HMC convergence metric and the model prediction by LOO cross-validation are affected by the level of prior information about global scaling parameter K. The model used in this study is a connectome-based model, thus its predictive power for seizure propagation depends critically on the estimation of the parameter K. Our results indicate that a (weakly) informative prior is required to obtain an accurate and reliable estimation in EZ/PZ locations. If we place an uninformative prior on this parameter (e.g., uniform), the HMC failed to converge. We now mentioned also this point in Results/Discussion. Another figure was added to appendix (Fig S2) to show the robustness of inversion for two other estimations of spatial map of epileptogenicity.

**Reviewer #2:** The paper builds on the authors’ prior efforts to develop dynamical systems model of large-scale brain network activity. Here, a specific application of these models is considered: the statistical testing of epileptogenic dynamics within specific brain regions. Using a ground truth model, the authors generate synthetic data of epileptic activity; then, a low-dimensional model is used to facilitate Bayesian inference of parameters associated with the disposition (epileptic or not) of different regions within the network. The main result pertains to the demonstration that fully Bayesian model selection and hypothesis testing criteria are more suitable to this inference problem versus popular techniques such as AIC or BIC.

We thank the reviewer for your review and the very constructive comments.

Main:
- In the primary finding, the authors generate 'ground truth' data from a high-dimensional dynamical neural model (the ‘virtual brain’); then they use Bayesian inferential techniques in order to perform hypothesis testing regarding the parameterization of the model based on observed model output. This inference is performed using a low-dimensional approximation of the model. In this setting, it is shown that fully Bayesian model selection techniques are able to adjudicate key parameters pertaining to nodal excitability in the full model. The issue as I see it is that the low dimensional model is obtained via formal dimensionality reduction of the high dimensional model, so it is relatively unsurprising that a Bayesian inferential technique will return high-quality parameter estimates in this setting. If the argument is that this work sets the stage for deploying this method on actual human data, then what one wants is a mis-matched ground truth model, wherein both the within- and across-node dynamics may deviate from those of the inference model. In other words, how would this inference technique perform if the "actual" brain deviated in its dynamics from those nominally embedded in the inference model?

We thank the reviewer for this comment, as the deployment on actual human data is indeed our motivation. We do not agree though that the matched ground truth model is straight forward to work. The Bayesian inversion of the reduced model is a very challenging task and to the best of our knowledge, there is no similar high-dimensional problem with nonlinear generative model in the literature of brain network modeling. In this study, by parcellating the brain into $N=84$ regions and having 1200 data points per region as the observation, the Bayesian inversion involves estimating 201600 hidden states and 225 free parameters ($k=3N+3$), which is very challenging to accurately estimate. Inference using a grid search or maximum likelihood estimation provide only a point estimation with no uncertainty in estimation and reliability in measuring model predictive power for unobserved data. In contrast, Bayesian framework deal with uncertainty in parameter estimation and allows us to integrate the prior information in model prediction. The fully Bayesian WAIC and LOO cross-validation used in this study summarize the out-of-sample model prediction power for unseen data through integrating individual data and calculating full posterior distributions to explain the fitted data. To clarify this point, in Introduction we have added:

“Large-scale brain network models emphasize the whole network character of the changes in the organization of brain activity as observed in brain disorders, which provide a useful link between brain dynamics in EZ (where the seizure starts) and the seizure propagation recruiting secondary subnetworks, the so-called Propagation Zone (PZ). While the location of the seizure onset area is usually invariant across seizures in an individual patient (Bartolomei et al. 2017, Proix et al. 2018), the PZ may be misclassified as the origin of pathological spatiotemporal patterns in pre-surgical monitoring. In the context of personalized brain modeling of the epileptic dynamics, the EZ/PZ prediction by a parametric approach such as the grid-based exploration of parameter space or Approximate Bayesian Computation (ABC)-related methods (Beaumont et al. 2002, Sunnaker et al. 2013) strongly relies on the distance measure used for model evaluation. Thereby, the in-sample prediction based on the summary statistic depends critically on the tolerance level in the accepted/rejected parameter setting. In contrast, the nonparametric Bayesian approach provides a robust and reliableprobabilistic framework for out-of-sample prediction. Moreover, it allows us to integrate patient-specific information such as brain connectivity as the prior knowledge as well as the uncertainty regarding the organization of EZ/PZ in the model likelihood function to improve out-of-sample prediction accuracy. Although an efficient Bayesian inversion requires painstaking model-specific tuning, the automatic probabilistic machine learning methods (Stan, PyMC3, Edvard, Pyro) featured by the self-tuning sampling algorithms (Hoffman et al. 2014, Carpenter et al. 2017) provide high-level flexible tools to solve complex inverse problems conditioned on large data set as the observation. Despite the theoretical and technological advances, practical implications of nonparametric fully Bayesian inference with information-theoretic measures for model selection to formulate and evaluate the clinical hypothesis on the location of EZ remain to be explored”.

We have added one figure in the revision (now Fig 5) to demonstrate the performance of the model and the limitation in Bayesian inversion. In this figure, we have shown how the accuracy, z_scores, HMC convergence metric, and the model prediction by LOO cross-validation are affected by the level of prior information about global scaling parameter K. The model used in this study is a connectome-based model, thus its predictive power for seizure propagation depends critically on the estimation of the parameter K. Our results indicate that a (weakly) informative prior is required to obtain an accurate and reliable estimation in EZ/PZ locations. If we place an uninformative prior on this parameter (e.g., uniform), then HMC failed to converge. We now mentioned this point in Results/Discussion. Another figure is added to the appendix (Fig S2) to show the robustness of inversion for two other estimations of spatial map of epileptogenicity. Regarding a mis-matched ground truth model, we added another figure in the appendix (now FigS4) to systematically show the estimation of excitability parameter for a selected brain region. This figure demonstrates how the model performs by the changes in within-node dynamics from those nominally values used in the manuscript. Please note that the spatial map of excitability across brain regions implies heterogeneity across-node dynamics, which we have shown for different configurations (see Fig 2C, and now Fig S2, panels A and B). As the within-node dynamics vary by the changes in excitability parameter, our results indicate an accurate and robust estimation by recovering the ground truth (FigS4, panel A). Since in our model the dynamics of EZ depend only on the excitability parameter, the model performance in EZ estimation remains accurate by the changes in the structural connectivity (SC) values (FigS4, panel B, shown in red). However, the seizure propagation depends also on network properties, thus the model performance in PZ estimation depends critically on the SC matrix. Consequently, by a dramatic change in the connections to these nodes, Bayesian inversion fails to correctly estimate the node dynamics in PZ.

Fig S4:

Estimation accuracy across different structural and functional components of the BVEP model. (A) Excitability values used for simulation versus the estimated values for a selected brain region (node number 6). As the node dynamics are varied by changing the excitability parameter (functional component), the model inversion by NUTS algorithm demonstrates an accurate and robust estimation by recovering the ground truth. Dashed red line represents a perfect fit. (B) The classification accuracy in EZ and PZ prediction as the value of structural connections (SC) to the selected brain region is decreased from 100% to 0% of value used in simulation (SC*), i.e. SC= (1-ε)SC*. As the dynamics in EZ depend only on the excitability parameter, the EZ prediction remains accurate irrespective of changes in SC (shown in red). However, the seizure propagation depends also on the network properties, thus the model performance in PZ prediction depends critically on the strength of connections to these nodes (shown in yellow).

- A key aspect of this pertains to assumptions regarding how structure/diffusion-based anatomical characterizations inform signal propagation throughout the network. Because these assumptions are essentially equivalent in the high-dimensional and low-dimensional model variants, it is entirely expected that predictions regarding signal transmission between nodes would be relatively consistent between the two. This also pertains to the results of Figure 5, which establishes that an individual’s structural connectivity parameterizing the low-dimensional model is best among a set of alternatives. At the point that the low-dimensional approximation is shown to be valid, I’m not sure what this added layer of Bayesian inference adds.

Thank you for mentioning this point. Since the BVEP is a personalized model of epilepsy, our results indicate that the fully Bayesian criteria such as WIAC and LOO cross-validation are able to correctly assess different hypotheses about both structural (individual’s structural connectivity) and functional components (excitability parameter) of the brain network models from observed data that differ across individuals. For instance, if we preserved the spatial map of excitability, the seizure propagation patterns will not be preserved for different patients and vice versa.

We agree with reviewer that the seizure initiation and the spatio-temporal propagation are invariant under model reduction, and the main motivation here is to reduce the computation time regarding model inversion while the spatial map of excitability is preserved. This point has been added in revision as follows:

“Note that, the full VEP model comprises five state variables governed by three different timescales (Eq. 1) and is a complete taxonomy of epileptic seizures, which demonstrate a thorough description of system bifurcations that give rise to onset, offset and seizure evolution (Jirsa et al. 2014, Saggio et al. 2020). Whereas, the 2D reduced variant of the model (Eq. 2) is limited to modeling the average of fast discharges during the ictal seizure states and the seizure propagation. This reduction is limited to demonstrate only saddle-node bifurcation out of six types of bifurcation involved in the spatio-temporal seizure dynamics. However, the seizure initiation from EZ and propagation to PZ are invariant under this model reduction (Proix et al. 2014, Proix et al. 2017). The 2D VEP model can successfully predict the key data features such as onset, propagation envelope and offset, while considerably alleviating the computational time of the Bayesian inversion (Jirsa et al. 2017, Hashemi et al. 2020).”

- Likewise, the demonstration of hypothesis testing on the single patient is conceptually problematic. It seems to me that the 'fitting' of the high dimensional model to the SEEG data is actually the key step here and this very poorly described in the paper (indeed, the entirety of the methods for this seem to be implied through a few lines in the results). If I understand it correctly, this fitting produces parameters in the high-dimensional model that reflect increased excitability in the Amygdala, which is then substantiated through the proposed hypothesis testing regimen. At the point that one has the high-dimensional model, then the hypothesis testing essentially amounts to the results of Figure 4. As such, I find this single-subject application to be quite superficial.

Thank you for mentioning this point. In order to avoid the non-identifiability for fitting the SEEG data, the contacts are selected according to the mean energy of bipolars to provide a bijection map between source activity (generated by Epileptor) at brain regions and measurement at electrodes. This point is now added in a subsection Stereotactic-EEG (SEEG) data preprocessing in Material/Methods. In addition, the codes and documentation for SEEG preprocessing is added in Github repository.

In this study we focused on synthetic data to validate the technical reliability of our approach, the self-tuning sampling algorithms (NUTS), and more importantly, the fully Bayesian information criteria and systematic cross-validation for measuring the out-of-sample prediction accuracy. Showing the estimations for the patient data requires a detailed non-trivial comparison with the clinical evaluations and outcome after surgery, involving significant other work such as group analysis (in particular homogenization of cohort), which was not aimed in this study. This remains to be critically investigated in future work.

Stereotactic-EEG (SEEG) data preprocessing:

For the selected patient, nine SEEG electrodes were placed in critical regions based on the presurgical evaluation. SEEG electrodes comprise 10 to 15 contacts. Each contact is 2 mm of length, 0.8 mm in diameter, and is 1.5 mm apart from other contacts. Brain signals were recorded using a 128-channel Deltamed™ system (sampling rate: 512 Hz, hardware band-pass filtering: between 0.16 and 97 Hz). The SEEG data is re-referenced using a bipolar montage, which is obtained using the difference of 2 neighboring contacts on one electrode. We extract the bipolarized SEEG signal from 5s before seizure onset up to 5s after seizure offset. The onset and offset times of the epileptic seizure are set by clinical experts. In this study, the log power of high frequency activity is used as the target for the fitting task. More precisely, the SEEG data are windowed and Fourier transformed to obtain estimates of their spectral density over time. Then SEEG power above 10 Hz is summed to capture the temporal variation of the fast activity (Jirsa et al. 2016, Proix et al. 2017). The envelope is calculated using a sliding-window approach with a window length of 100 time points. The signal inside the window is squared, averaged and log transformed. From the resulting envelope, we again identify and remove outliers as described above. Finally, the envelope is smoothed using a lowpass filter with a cut-off in the range of 0.05 Hz. The mean across the first few seconds of the envelope is used to calculate a baseline which is then subtracted from the envelope. Contacts are selected according to the mean energy of bipolars to provide a bijection map between source activity at brain regions and measurement at electrodes.

- At a high level, it is unclear to me that PloS Computational Biology is the right forum for this paper. The biological insight conferred by these results is minimal at best; the primary contribution is in the space of methodological enhancement. That the fully Bayesian or LOO methods outperform BIC or AIC is a reasonable and potentially useful finding, but it is also quite narrow in scope given above considerations. Indeed, as shown in Figure 4, BIC or AIC still "work" albeit with a lower level of statistical ambiguity.

Please note that while using AIC/BIC for hypothesis testing, there is still a strong support for the alternative hypotheses, since they are defined based on uniform priors and single point-estimation, they are not able to provide significant evidence for the correct (ground-truth) hypothesis. If we do not truncate the prior, AIC/BIC are not able to distinguish different epileptogenicity hypothesis since the deviance and penalty terms are identical for each of epileptogenicity hypothesis (the number of data and model parameters are identical for different epileptogenicity hypotheses). In appendix (Fig S4), we have shown that there is no significant difference between different hypotheses for 100 runs. In contrary to classical information criteria, fully Bayesian information criteria such as WAIC and LOO correctly favor the true hypothesis with a high level of statistical significance.

Since the Bayesian inversion and model selection is a critical task in mathematical modeling of biological systems, we believe that this work will be of interest to the PLOS CB readers. To provide better context to the readers, now Introduction begins as:

“Mathematical models have long played a key role in understanding biological systems. However, the intrinsic uncertainty associated with parameters of dynamical models translates into an uncertainty in model predictions, thereby in the selection among a set of candidates that best balance between complexity and accuracy. In practice, the nonlinearity and complexity of most dynamical models render the model calibration and hypothesis testing nontrivial (Raftery 1995, Piironen et al. 2017, Hashemi et al. 2018, Hashemi et al. 2020). Bayesian frameworks offer a powerful approach to deal with model uncertainty in parameter estimation and a principled method for model prediction with a broad range of applications (Friston et al. 2003, Kiebel et al. 2009, Daunizeau et al. 2014, Cooray et al. 2015, Cooray et al. 2016, Coelho et al. 2011, Lillacci et al. 2010, Sun et al. 2008, Jirsa et al. 2017). Bayesian techniques infer the distribution of unknown parameters of the underlying data generating process given only observed responses and prior information about the underlying generative process (Gelman et al. 1995, Bishop 2006). Thus, the estimated posterior is dependent on the level of information in the prior distribution, as well as the conditional probability of the observed data given the model parameters in the form of likelihood function. Monte Carlo Markov Chain (MCMC) methods are able to calculate the unnormalized posterior densities (Gelman et al. 1995, Brooks et al 2011). However, MCMC-based sampling of posterior distributions, which converge to the desired target distribution for large enough sample size, is a challenging task due to the large curvature in the typical set (Betancourt 2013b, Betancourt 2014) or non-linear correlations between parameters in high-dimensional parameter spaces (Hoffman and Gelman 2014, Betancourt 2016a, Betancourt 2018). ”

- There are many grammatical issues throughout the paper that impede readability. A thorough round of copyediting is needed.

Thank you for this remark. We have now improved the manuscript in terms of language editing.

Minor:

- This is a stylistic qualm, but I feel that the first part of the Introduction is far too technical without providing any context as to the points about model selection, information criteria etc. If would be helpful to first discuss the models being considered, their goals and then move into the problem at hand. I had to read through to the results before I started understanding what the paper was trying to accomplish.

Thank you for mentioning this point. As mentioned above, Introduction has been reorganized and we have provided more context for the readers.

- Is there a reason the authors choose epileptic zone, as opposed to the clinical term of an epileptic focus?

Yes. Today the expression epileptogenic zone acknowledges the fact that there is a significant spatial extent of epileptogenicity. This point has been added in revision as follows:

“The success rate of epilepsy surgery averages only about 50-60% and has not progressed in 30 years (Cloppenborg et al. 2016, Kuhlmann et al. 2018). Successful outcomes of surgical interventions depend critically on the ability to accurately identify the Epileptogenic Zone (EZ) in the brain, and the subsequent propagation to other close or distant brain regions. In this study, the EZ is defined as the pathological area(s) in the brain responsible for the origin and early organization of the epileptic activity (Proix et al. 2017). Following previous studies, the term epileptogenic zone is used rather than the term epileptic focus to better describe the complexity and often spatially distributed nature of seizure initiation in the brain, not restricted to a single focus (Talairach et al. 1996, Bartolomei et al. 2008, Bartolomei et al. 2017).”

- P8l102: probabilistic brain network approach--> modeling approach?

This has been fixed.

- Figure 4 and Table 1 seem quite redundant.

For better communication and ease of retrieval of information, we would like to retain Table 1, since it reports the detailed results related to figure.

- The use of ‘virtual brain model’ in the abstract is awkward. A model is by definition ‘virtual’ insofar as it is not the actual system at hand. Adding ‘virtual’ seems superfluous.

The expression “virtual brain” has become a commonly, albeit loosely, used terminology for full brain modeling using connectomes and neural mass models at network nodes. Historically, this derives from the use of the neuroinformatics platform The Virtual Brain (<https://www.thevirtualbrain.org>).

**Reviewer #3**: In this paper Hashemi et al. provide a study of how different strategies for model comparison to infer node features in an epileptic network using the 'virtual epileptic patient' framework. The work represents a much needed step towards integrating empirical (clinical) data into modelling approaches and illustrates nicely many key issues and a few crucial insights. Whilst I think that it would benefit from addressing some major concerns, I belief overall the work represents an important contribution with valid insights.

We are grateful to the reviewer for the interest in our work and for the positive and very helpful suggestions.

Major
====
- The authors show a single example of two types of inference (one pertaining to node-identity, one pertaining to structural connectivity identity). Are these representative? Were these the first ones tried? Does the inference break down? If so, where?

In this study, we have assumed that the seizure initiation in EZ depends only on the excitability parameter of the brain regions. If the Epileptors are disconnected (i.e., K = 0; no network coupling), the seizures are triggered only in regions specified as EZ. By coupling the Epileptors through the patient-specific structural connectivity matrix (K >0), depending on the values of excitabilities, the noise level, and the connectivity strength, the seizures may propagate to the candidate brain regions defined as PZ.

We have added one figure in the revision (now Fig 5) to demonstrate the performance of model and the limitation in Bayesian inversion. In this figure, we have shown how the accuracy in EZ/PZ prediction, the z_scores, the HMC convergence metric and the model prediction by LOO cross-validation are affected by the level of prior information about global scaling parameter K. The model used in this study is a connectome-based model, thus its predictive power for seizure propagation depends critically on the estimation of the parameter K. Our results indicate that a (weakly) informative prior is required to obtain an accurate and reliable estimation in EZ/PZ locations. If we place an uninformative prior on this parameter (e.g., uniform), the HMC failed to converge. We now mentioned also this point in Results/Discussion. Another figure was added to appendix (Fig S2) to show the robustness of inversion for two other estimations of spatial map of epileptogenicity.

In Discussion we have mentioned:

“Combining individual's anatomical information with mean-field models of abnormal neural dynamics, in this study, the seizure initiation was defined as an intrinsic brain region property, whereas the seizure propagation was characterized by complex spatiotemporal dynamics of large-scale brain network. In other words, we assume that the seizure originates from a local network and recruits candidate brain region(s) strongly coupled to the pathological areas by perturbing their stable dynamics (if $K = 0$, then there is no seizure recruitment). In connectome-based modeling approach, the model predictive power can then dramatically depend on the estimation of the global coupling parameter ($K$), which scale different region's influence in perturbing the emergent dynamics of a selected region through structural connectivity (SC). An overestimation of $K$ leads to misclassification of PZ as HZ, whereas an underestimation of coupling may yield to misclassification of PZ as EZ. We observed that for convergence of BVEP model, an informative or a weakly informative prior is required on the parameter $K$, whereas using an uninformative prior the HMC chains failed to converge (cf. Fig 5).

- Underlying assumptions regarding ictogenesis in the epileptic brain: The approach presented here seems to make strong assumptions regarding the generation of seizures in the epileptic brain: As presented here seizures arise from the focally abnormal activity of individual nodes and connectivity only describes their subsequent spread. Seizures could therefore never be said to arise from the interaction of two (individually not 'EZ' nodes)? Is this an assumption the authors make? Could they clarify this in the text?

We thank reviewer for mentioning this point. As mentioned correctly, in this work, the seizure recruitment is characterized by complex spatio-temporal dynamics of large-scale brain network i.e., seizure originates from a local network and recruits candidate brain regions strongly coupled to the pathological areas by perturbing their stable dynamics (if K = 0, the seizures can trigger but there is no propagation).

This point is now clarified in Material/Methods as:

“Here, we assume that seizures originate from a subnetwork(s) denoted by EZ, and recruit a secondary subnetwork(s) denoted by PZ that are strongly coupled to the EZ. In other words, seizure initiation is a region's intrinsic property and depends only on the excitability parameter of the brain regions, whereas seizure propagation is a complex network effect which depends on the interplay between multiple factors including the brain region's excitability (node dynamics) (Bartolomei et al. 2008, Goodfellow et al. 2016, Lambert et al. 2018), the individual structural connectivity (network structure) (Proix et al. 2017, Proix et al. 2018, Olmi et al. 2019), and brain state dependence (network dynamics).”

- There is clearly the potential for integration of this work into the clinic, and the desire of the authors to illustrate this potential. For this it would be helpful which hypothesis the approach could test, and which part of the presented analysis speak to these pre-defined types of hypotheses that need testing. How does e.g. the identification of patient specific S.C. matrices relate to clinical applicability? Is it just a validity demonstration? What validity does this show?

We thank reviewer for mentioning the positive comment. Since the model relies on the structural data of individuals and presented as a personalized brain model of epilepsy, we showed that the fully Bayesian information criteria and LOO cross-validation are able to assess different hypotheses about both structural (patient connectome) and functional (region excitability) components of whole-brain network models that differ across individuals. Our results can illustrate the ability of large-scale brain modeling approach to better understand the pathophysiology of brain disorders for personalized medicine. Superior predictive power of use of individual connectomes has been demonstrated in detailed rodent studies by Melozzi et al (PNAS, 2020). This demonstrates so-called internal validity. External validity has previously been demonstrated by Proix et al (2018) in retrospective patients, whereas a currently ongoing trial (EPINOV, coordinator Fabrice Bartolomei) tests external validity in prospective patients.

- The limitations of the model are not discussed sufficiently. Two apparently selective inference examples are shown without clear discussion as to where the limits of inference on this very high dimensional brain models would be. Examples of how then inference breaks down and where it is applicable would be useful.

We thank reviewer for mention this important point. As mentioned above, one figure was added to appendix (Fig S2) to show the robustness of inversion for two other estimations of spatial map of epileptogenicity. We have shown that by placing a weakly informative prior on global scaling parameter K, the HMC is able to accurately estimate the brain nodes excitability parameter for different configurations of EZ/PZ.

We have added one figure in the revision (now Fig 5) to demonstrate the performance of model and when the HMC sampling failed to converge. The limitation in Bayesian inversion of model is also now mentioned in Discussion.

Minor
====
- The manuscript would benefit from some (light touch) English language editing for readability. Furthermore there are several typos throughout the manuscript that could be corrected for the next submission ('self-tuninginvariant', 'Structrural brain network model', 'predict the envelop' etc.).

Thank you for mentioning these typos. They have been corrected. We have also improved the manuscript in terms of language editing.

- Introduction: Why is epilepsy an appropriate model for the verification of this approach. Since the gold standard of empirical seizure progression is not easily knowable, why not choose to fine tune the inference strategies for these whole-brain models on more constrained empirical data?

Following reviewer comment, this point has been added in Introduction:

“The success rate of epilepsy surgery averages only about 50-60% and has not progressed in 30 years (Cloppenborg et al. 2016, Kuhlmann et al. 2018). Successful outcomes of surgical interventions depend critically on the ability to accurately identify the Epileptogenic Zone (EZ) in the brain, and the subsequent propagation to other close or distant brain regions. In this study, the EZ is defined as the pathological area(s) in the brain responsible for the origin and early organization of the epileptic activity (Proix et al. 2017). Following previous studies, the term epileptogenic zone is used rather than the term epileptic focus to better describe the complexity and often distributed nature of seizure initiation in the brain, not restricted to a single focus (Talairach et al. 1996, Bartolomei et al. 2008, Bartolomei et al. 2017).”

“Large-scale brain network models emphasize the whole network character of the changes in the organization of brain activity as observed in brain disorders, which provide a useful link between brain dynamics in EZ (where the seizure starts) and the seizure propagation recruiting secondary subnetworks, the so-called Propagation Zone (PZ). While the location of the seizure onset area is usually invariant across seizures in an individual patient (Bartolomei et al. 2017, Proix et al. 2018), the PZ may be misclassified as the origin of pathological spatiotemporal patterns in pre-surgical monitoring. In the context of personalized brain modeling of the epileptic dynamics, the EZ/PZ prediction by a parametric approach such as the grid-based exploration of parameter space or Approximate Bayesian Computation (ABC)-related methods (Beaumont et al. 2002, Sunnaker et al. 2013) strongly relies on the distance measure used for model evaluation. Thereby, the in-sample prediction based on the summary statistic depends critically on the tolerance level in the accepted/rejected parameter setting. In contrast, the nonparametric Bayesian approach provides a flexible probabilistic framework for out-of-sample prediction. Moreover, it allows us to integrate patient-specific information such as brain connectivity as the prior knowledge as well as the uncertainty regarding the organization of EZ/PZ in the model likelihood function to improve out-of-sample prediction accuracy. Although an efficient Bayesian inversion requires painstaking model-specific tuning, the automatic probabilistic machine learning methods (Stan, PyMC3, Edvard, Pyro) featured by the self-tuning sampling algorithms (Hoffman et al. 2014, Carpenter et al. 2017) provide high-level flexible tools to solve complex inverse problems conditioned on large data set as the observation. Despite the theoretical and technological advances, practical implications of nonparametric fully Bayesian inference with information-theoretic measures for model selection to formulate and evaluate the clinical hypothesis on the location of EZ remain to be explored”.

- Regarding the model parameters: Fig 2E: Please elaborate on what the 200,000 model parameters are, how many of these are free to vary during model inference and how this apparently huge parameter space can be constrained by very limited datasets

Thank you for mentioning this point. The figure label has been corrected to avoid the ambiguity. We have mentioned this point in Results:

Here, by parcellating the brain into $N=84$ regions and having 1200 data points per region as the observation, the Bayesian inversion involves estimating 201600 hidden states and 225 free parameters ($k=3N+3$).

It has been shown previously that that NUTS algorithm is able to efficiently sample from high-dimensional target distributions that allow us to solve complex inverse problems conditioned on nonlinear generative models and large data set as the observation (Gopalan and Blei, 2013; Gopalan et al., 2016; Kucukelbir et al., 2017).

- Regarding the S.C. reconstructions - there is a non-trivial link between structural connectivity (of any kind, but particularly of those measured with DTI-based imaging methods) and effective synaptic connectivity. Is this link mediated through a fitted parameter?

If the notion of effective synaptic connectivity indicates estimated causal interactions (Friston Brain Connectivity 2011) in synaptic weights, the target of our fitting (epileptogenicity parameter) is not able to explain the direct influence that one neuronal population exerts on another. However, the changes in this parameter are associated with increased seizure susceptibility, as it alters synaptic transmission (Jirsa et al, Brain 2014). Effective synaptic connectivity was beyond the scope of this study and remains to be investigated in future work.

- More clinical context regarding the patient could be valuable if possible to include (particularly syndromic epilepsy diagnosis, final histological diagnosis after operation)

This point has been added in subsection Individual patient data:

For this study, we selected a patient (a 35 year-old male) initially diagnosed with left temporal lobe epilepsy (Histopathology: Gliosis, Surgical procedure: resection, Surgical outcome: seizure free, Engel score I).

- Is the approach presented here sensitive to changes in Atlas resolution? How was the specific Atlas resolution utilised here chosen.

In this study, we used Desikan-Killiany parcellation in the reconstruction pipeline, and the patient’s brain is divided into 68 cortical regions and 16 subcortical structures. In an ongoing clinical trial, we are using VEP atlas (Huifang et. al Journal of Neuroscience Methods,

2020) with 162 brain regions (including 73 cortical and 8 subcortical regions per hemisphere) and the algorithm is still able to converge.

- for those not familiar with the tools, it might be worthwhile to introduce the relevant 'PPL' tools and cite their relevant references where available

Thank you for mentioning this point. This has been added to Material/methods:

Stan is a state-of-the-art platform for statistical modeling and high-performance statistical computation which can be run in popular computing environments (see https://mc-stan.org). PyMC3 provides several MCMC algorithms for model specification directly in Python code (see https://docs.pymc.io).

- Are there examples of where the inference algorithms fail and do not produce the expected posterior shrinkage

The VEP model is a connectome-based model, and its performance for prediction of seizure propagation depends critically on the estimation of the global coupling parameter K. Our results indicate that a (weakly) informative prior is required to obtain an accurate and reliable estimation in EZ/PZ locations. If we place an uninformative prior on this parameter (e.g., uniform), then HMC failed to converge.

- a substantial amount of the paper focusses on the interesting and relevant developments in sampling algorithms, measures for model comparison, and computing structure, which is only alluded to in the introduction

Following reviewer comment, Introduction has been reorganized to address this point.

- for choice of EZ / PZ in the simulation, was existing connectivity between nodes taken into account or matched to empirical data somehow. I notice that these were spatially quite segregated in the model (but it is not clear whether they are in fact strongly connected or not). Is this representative of real patient data? I would have anticipated PZ and/or EZ nodes to be fairly well connected to each other and/or spatially contiguous

Thank you for mentioning this point. In this work, the seizure recruitment is characterized by complex spatiotemporal dynamics of large-scale brain network i.e., seizure originates from a local network and recruits candidate brain regions strongly coupled to the pathological areas by perturbing their stable dynamics.

The choice of EZ/PZ was based on experimental findings, however, since the seizures propagation to PZ is a network property, we selected the nodes that are strongly coupled to EZ for recruitment. Among the candidate brain regions for seizure propagation, due to stronger connection to the pathological areas defined as EZ, a few of nodes can be recruited by a weak global coupling. Rather, a stronger coupling with high node excitability is required for seizure recruitment to all other candidate brain areas. This is in agreement with experimental observations that seizures tend to have a common spatial origin in the same patient (Proix et al., 2017, 2018). This point is now added in revision:

Here, we assume that seizures originate from a subnetwork(s) denoted by EZ, and recruit a secondary subnetwork(s) denoted by PZ that are strongly coupled to the EZ. In other words, seizure initiation is a region's intrinsic property, whereas seizure propagation is a complex network effect which depends on the interplay between multiple factors including the brain region's excitability (node dynamics) (Bartolomei2008, Goodfellow2016, Lambert2018), the individual structural connectivity (network structure) (Proix2017, Proix2018, Olmi2019), and brain state dependence (network dynamics).

- different imaging modalities are frequently alluded to - I appreciate that this is relevant for the wider applications of TVB, but it is not clear how other imaging modalities (especially fMRI) will have a clear role in the near future for inference of ictal brain dynamics as presented here

Other imaging modalities can provide us more level of information about the brain region property to increase model predictive power by their integration in prior information. To address this question, we have added this point to Discussion:

Other brain imaging modalities such as fMRI/MEG can provide more information about the excitability of brain regions (Pizzo et al. 2019, Courtiol et al. 2020) or global scaling parameter (Schirner et al.2018, Deco et al. 2019). However, fitting simultaneous brain recordings with different spatial and temporal resolutions is more challenging.

Figure 2: Model fits vs Observation - indeed the fit seems very good and I am somewhat surprised that the fitted and the observed data 'start' seizures at the same time.
- is there any noise driving the system?

In PZ, the excitability is close to the critical value and for weakling global coupling, a sufficient amount of noise is required for seizures propagation. In the simulation, a zero-mean white Gaussian noise with a variance of 0.0025 was added to the system state variables.

- what defines the point of seizure onset? Will every simulation produce a seizure at the same time (because of e.g. constant rate input current?). How are the initial states of the system defined (e.g. of the slow variables)? Are they fitted as well?

In simulation, the initial conditions were selected randomly in the interval $(-2.0, 5.0)$ for each state variable, and a weakly informative prior with the mean at ground-truth and standard deviation of 1.0 are placed and for inference. The seizure initiation in EZ is independent of the noise and coupling, whereas the propagation depends on coupling and noise, thus its starting point may vary for different realizations. The fitting target is raw time series of only fast variable (x_1 in Eqs. (1) and (2)), whereas the inferred hidden states include the slow variable z also. In other words, we inferred a slow-fast system in 2D reduction, while the time scale ($\tau$) was fixed as a constant value. The inference showed a very good agreement between the simulated and inferred limit cycles corresponding to EZ/PZ. An exemplary of code in Jupyter notebook showing the inferred dynamics in phase-plane is available at Github repository.

Figure 3: Colour bar axes would be helpful.

The figure is illustrated with color bar axes.

How do Epileptor model states relate to SEEG data? Is there additional conversion or are they taken to just represent the log of the raw SEEG signal? What montage is that signal calculated from? Does that have an impact on the inversion?

Thank you for mention this point. In order to avoid the non-identifiability for fitting the SEEG data, the contacts are selected according to the mean energy of bipolars to provide a bijection map between source activity (generated by Epileptor) at brain regions and measurement at electrodes. This point is now added in a subsection Stereotactic-EEG (SEEG) data preprocessing in Material/Methods. In addition, the codes and documentation for SEEG preprocessing was added Github repository.

Reviewer #3: **No:** - numerical results from simulation on which inference is built not available

The source codes related to forward simulation in TVB and SEEG preprocessing along with more documentation have been added to Github repository.
